# Behavioral effects of continuous theta-burst stimulation in macaque parietal cortex

**DOI:** 10.1038/s41598-021-83904-8

**Published:** 2021-02-24

**Authors:** Lara Merken, Marco Davare, Peter Janssen, Maria C. Romero

**Affiliations:** 1 Laboratory for Neuro- and Psychophysiology, KU Leuven, 3000 Leuven, Belgium; 2 College of Health and Life Sciences and Centre for Cognitive Neuroscience, Brunel University London, UxBridge, UB8 3PN UK; 3 Leuven Brain Institute, KU Leuven, 3000 Leuven, Belgium

**Keywords:** Neuroscience, Psychology

## Abstract

The neural mechanisms underlying the effects of continuous Theta-Burst Stimulation (cTBS) in humans are poorly understood. Animal studies can clarify the effects of cTBS on individual neurons, but behavioral evidence is necessary to demonstrate the validity of the animal model. We investigated the behavioral effect of cTBS applied over parietal cortex in rhesus monkeys performing a visually-guided grasping task with two differently sized objects, which required either a power grip or a pad-to-side grip. We used Fitts’ law, predicting shorter grasping times (GT) for large compared to small objects, to investigate cTBS effects on two different grip types. cTBS induced long-lasting object-specific and dose-dependent changes in GT that remained present for up to two hours. High-intensity cTBS increased GTs for a power grip, but shortened GTs for a pad-to-side grip. Thus, high-intensity stimulation strongly reduced the natural GT difference between objects (i.e. the Fitts’ law effect). In contrast, low-intensity cTBS induced the opposite effects on GT. Modifying the coil orientation from the standard 45-degree to a 30-degree angle induced opposite cTBS effects on GT. These findings represent behavioral evidence for the validity of the nonhuman primate model to study the neural underpinnings of non-invasive brain stimulation.

## Introduction

Transcranial Magnetic Stimulation (TMS) is widely used to modulate brain activity in healthy volunteers and patients^1–7^. While a single TMS pulse can activate neurons (and induce a muscle twitch when applied over the primary motor cortex), repetitive TMS protocols can either temporarily increase or decrease neuronal excitability. Huang et al.^8^ described a reduction in the Motor Evoked Potential (MEP) after continuous Theta-Burst Stimulation (cTBS) over primary motor cortex, in which 50 Hz triplets of TMS pulses were administered every 200 ms (5 Hz) for 20 to 40 s. Since this seminal study, numerous publications have used cTBS as a tool to reduce cortical excitability and further investigate cTBS behavioral effects in humans^9–13^.

Despite a vast body of TMS research, very little is known about the neuronal effects of this noninvasive neuromodulation technique, which is partially due to the limited number of experimental models and tools explored until recent years. Previous TMS research has been mainly performed in combination with functional Magnetic Resonance Imaging (fMRI) and electroencephalography (EEG) in humans. Because these two techniques provide indirect measurements of neural activity, an animal model in which researchers apply TMS during invasive extracellular recordings was necessary. Mueller et al.^14^ recorded action potentials in awake monkeys shortly after the TMS burst. We recently charted the effect of single-pulse TMS on individual neurons in parietal cortex while monkeys were performing a grasping task^15^. TMS evoked a short burst of activity in single neurons, but the volume of cortex in which it induced a significant response was surprisingly small (2 by 2 by 2 mm). Moreover, the activation caused by TMS was frequently followed by reduced activity in task-related neurons, which was paralleled by a significant increase in grasping time (GT).

The nonhuman primate (NHP) model therefore provides significant advantages compared to other animal models for the study of TMS effects on neural activity. The presence of sulci and gyri—similar to the human brain and unlike the brains of rodents—determines the current spread^16^ and thereby the size of the activated area. In addition, using NHPs we can test TMS effects in much more controlled conditions, which are more difficult to achieve in human volunteers. For example, the TMS coil can be rigidly positioned on the skull, at exactly the same location from day to day and with the same angle^15^, by anchoring it to a pair of rods previously implanted on the head of the animal, avoiding several potential sources of variability such as subtle differences in coil positioning. More importantly, NHPs can perform a number of motor tasks, which allows studying the effects of TMS on both neurons and behavior, so that the results can be compared with studies in humans. Similar to the use of functional Magnetic Resonance Imaging (fMRI) in monkeys and humans, it is essential that the same measurement is applied in the two species, since any discrepancy between results obtained in humans and in NHPs could be due to either a species difference or a difference in the measurement technique (behavior or single-cell responses). Thus, prior to electrophysiological recordings during cTBS in NHPs, it was necessary to investigate the behavioral effects—magnitude, time course and the object specificity—of cTBS in the NHP model.

Significant progress in our understanding of the effects of cTBS on the human brain critically depends on a valid animal model, in which we can combine behavioral and physiological measurements^17^. Therefore, before investigating the effect of cTBS on single neurons, our goal was to study the behavioral effects of cTBS applied over parietal cortex in macaque monkeys. Notably, we wanted to verify whether cTBS induces a behavioral deficit with a similar time course as in humans. Moreover, we wanted to determine whether interfering with activity in PFG disrupted normal visuomotor processing of object size. We used a visually-guided grasping task in which monkeys had to grasp either a large object (requiring power grip) or a small object (requiring pad-to-side grip). This design allowed us to investigate cTBS effects on the GT for different object sizes, where Fitts’ law^18^ predicts shorter GTs for large compared to small objects. Before cTBS, monkeys’ GTs were indeed faster when grasping the large compared to the small object. However, after cTBS, GTs became longer for the large object and shorter for the small object, so that this GT difference was abolished. Thus, cTBS induced object-specific changes in GT. The temporal dynamics of these behavioral effects were consistent with those described in human volunteers but remained present for up to two hours (a long period not usually tested in humans). We conclude that cTBS applied over the NHP parietal cortex induces robust, object-specific and long-lasting motor deficits.

## Results

### Baseline grasping times validating Fitts’ law

**Figure 1:**
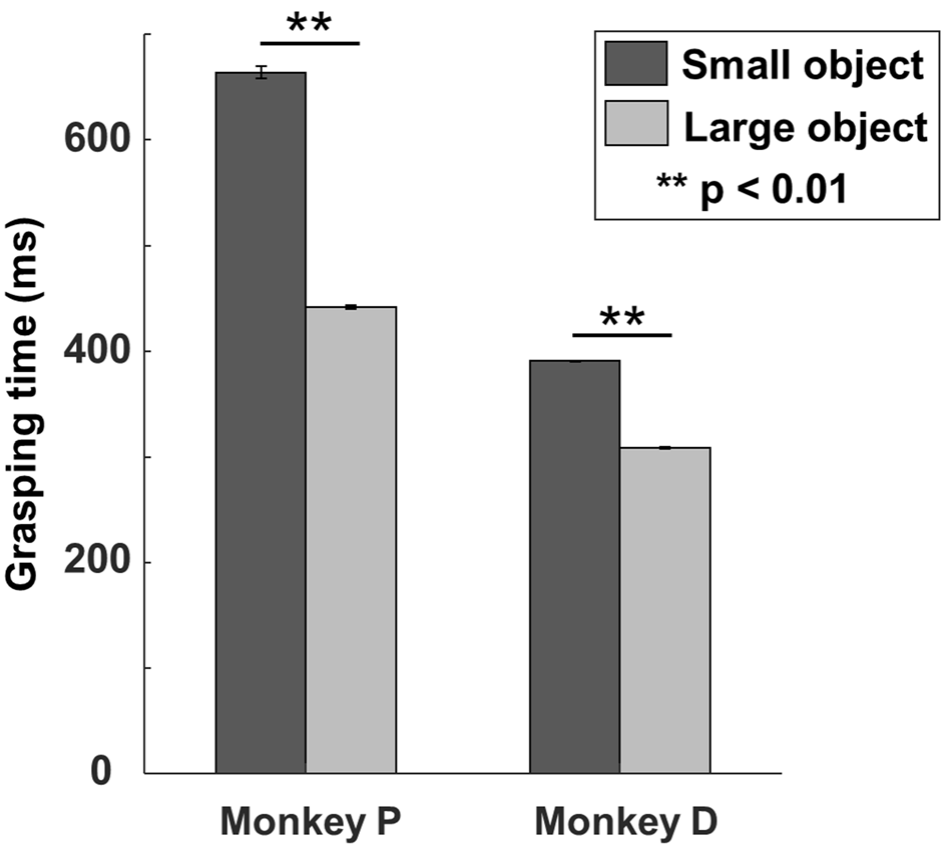
Baseline grasping times. Bar plot representing the GTs measured for each monkey, separately during the 4 baseline sessions performed with the small (black) and the large object (grey). Our data confirmed Fitts’ law, showing significantly longer GTs when grasping the small object. Asterisks indicate statistical significance (two-sided Wilcoxon Ranksum test; **: *p* < 0.01).

We first investigated whether we could observe Fitts’ law effects on our monkeys’ grasping behavior in absence of cTBS. In the baseline sessions (see Table 1 for details), both monkeys needed significantly more time to grasp the small object than the large object (Fig. 1, Wilcoxon rank sum test, *p* < 0.0001 for both monkey P and D). Moreover, the ratio of the average GT for the small object to the average GT for the large object (1.38 averaged for the two monkeys; 1.50 for monkey P and 1.26 for monkey D) was comparable to the ratio of the index of difficulty (ID = 1.40, see Methods). Thus, in line with an earlier study^19^, the behavioral performance of our monkeys followed Fitts’ law. Note, however, that monkey D was considerably faster in grasping the two objects than monkey P.

**Table 1:** Total number of sessions and correct trials collected during the baseline experiment.

Object type	Number of sessions	Number of correct trials	Total number of trials
Monkey P			
Large object	4	3422	4043
Small object	4	3120	4020
Monkey D			
Large object	4	4025	4826
Small object	4	3679	4526

### cTBS effect on grasping time

**Figure 2:**
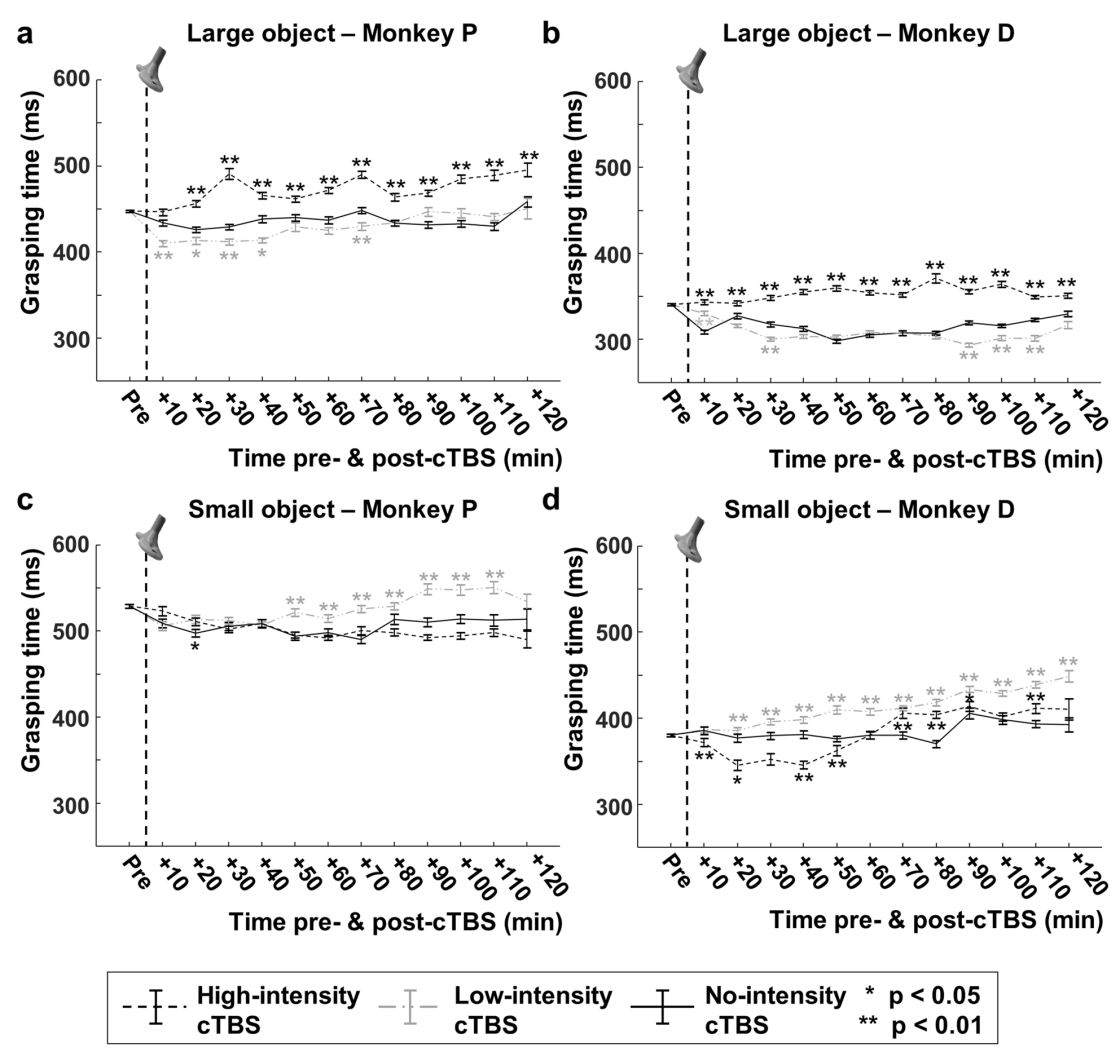
cTBS effect on grasping time. (**a**, **b**) Normalized grasping times for high- (dashed black line), low- (dashed grey line) and no-intensity (solid black line) cTBS as a function of time, for the large object for monkey P (**a**) and monkey D (**b**). Grasping times of minimally 1700 trials were averaged in time-epochs of 10 min. (**c**, **d**) Grasping times for the small object for monkey P (**c**) and D (**d**); same conventions as in (**a**, **b**). Asterisks indicate statistical significance (two-sided Wilcoxon Ranksum test, *: *p* < 0.05, **; *p* < 0.01). The vertical dashed line represents the stimulation time (cTBS).

The application of cTBS caused highly significant effects on GT in both animals (Kruskal–Wallis one-way ANOVA on the entire post-cTBS interval for the three stimulation intensities high-, low- and no-stimulation or HS, LS and NS; Large object monkey P: H = 867.28, *p* = 4.70e^−189^ , df = 2; Small object monkey P: H = 284.47, *p* = 1.69e^−62^, df = 2; Large object monkey D: H = 1900.16, *p* < 0.0001, df = 2; Small object monkey D: H = 763.9, *p* = 1.32e^−166^ , df = 2). Because studies in humans^8^ have demonstrated that the effect of cTBS increases over time, we plotted the GTs for the large and small object post-cTBS in our three stimulation conditions (no-, low- and high-intensity stimulation; Fig. 2). In both animals, HS-cTBS caused a robust and significant increase in GT for the large object (Fig. 2a,b), which grew over time and peaked 30 min post-cTBS in monkey P (Fig. 2a) and 80 min post-cTBS in monkey D (Fig. 2b; repeated measures ANOVA interaction between *time* and *stimulation intensity*, F(12,1584) = 4.62, *p* = 9.918^−11^, ${{\eta}}_{\mathrm{p}}^{2}$ = 0.066 for monkey P and F(12,2664) = 24.054, *p* = 4.071e^−74^, ${{\eta}}_{\mathrm{p}}^{2}$ = 0.178 for monkey D). At the peak of the effect, the GT had increased by 62 ms (+ 14.4%) and 64 ms (+ 20.9%) in monkey P and monkey D, respectively, compared to no-stimulation sessions. However, after this peak effect, the GT continued to differ significantly in high-stimulation sessions compared to no-stimulation sessions, with a significant increase in GT of 45 ms (+ 10%, monkey P) and 21 ms (+ 6.5%, monkey D) in the last time epoch measured (120 min post-cTBS). The effect of HS-cTBS on GT was very different for the small object (Fig. 2c,d). After a small initial increase (20 min post-cTBS), the GT of monkey P tended to become shorter (90–100 min post-cTBS, 19 ms, − 3.6%) compared to no-stimulation trials (Fig. 2c). In monkey D, a significant shortening of GT occurred 10–50 min post-cTBS (− 6.4% on average, *p* < 0.01 40–50 min post-cTBS), after which the GTs increased again and became longer than in no-stimulation trials (Fig. 2d).

Unexpectedly, the effect of LS-cTBS was (with the exception of the last time epochs for the small object in monkey D) almost always opposite than that of HS-cTBS. Indeed, at least in a number of time epochs, LS-cTBS gave rise to shorter GTs for the large object, and longer GTs for the small object (Fig. 2). For the large object (power grip), we measured on average a 4.0% reduction in GT in the 10–70 min post-cTBS epoch in monkey P (Fig. 2a), and a 5.4% reduction in GT in the 30 min post-cTBS epoch in monkey D (Fig. 2b; and an 8% reduction at 90 min). For the small object (pad-to-side grip), in contrast, the GT rose by + 5.5% in the 50–120 min post-cTBS epoch in monkey P (Fig. 2c) and + 8.1% in the 20–120 min post-cTBS epoch in monkey D (Fig. 2d). Thus, cTBS induced dose-dependent and grasp-specific effects on the GTs of both animals. In contrast, cTBS did not significantly affect the proportion of error trials (Table 3, z-test for proportions, all *p*-values > 0.05).

We investigated whether HS-cTBS also affected GTs in individual sessions by comparing the average GTs at the peak of the effect with those measured in the pre-cTBS interval, and comparing these with no-stimulation sessions. Because the effects we observed were strongest for the large object, we focused on the cTBS sessions with the large object. We measured significant increases in GT in every HS-cTBS session (N = 12), and the effect sizes were larger compared to NS-cTBS in 10/12 sessions. The time of the maximum effect for the large object occurred after 30 min (session 1), 30 min (session 2) and 100 min (session 3) in monkey P, and after 80 min (session 1), 80 min (session 2) and 120 min (session 3) in monkey D.

**Figure 3:**
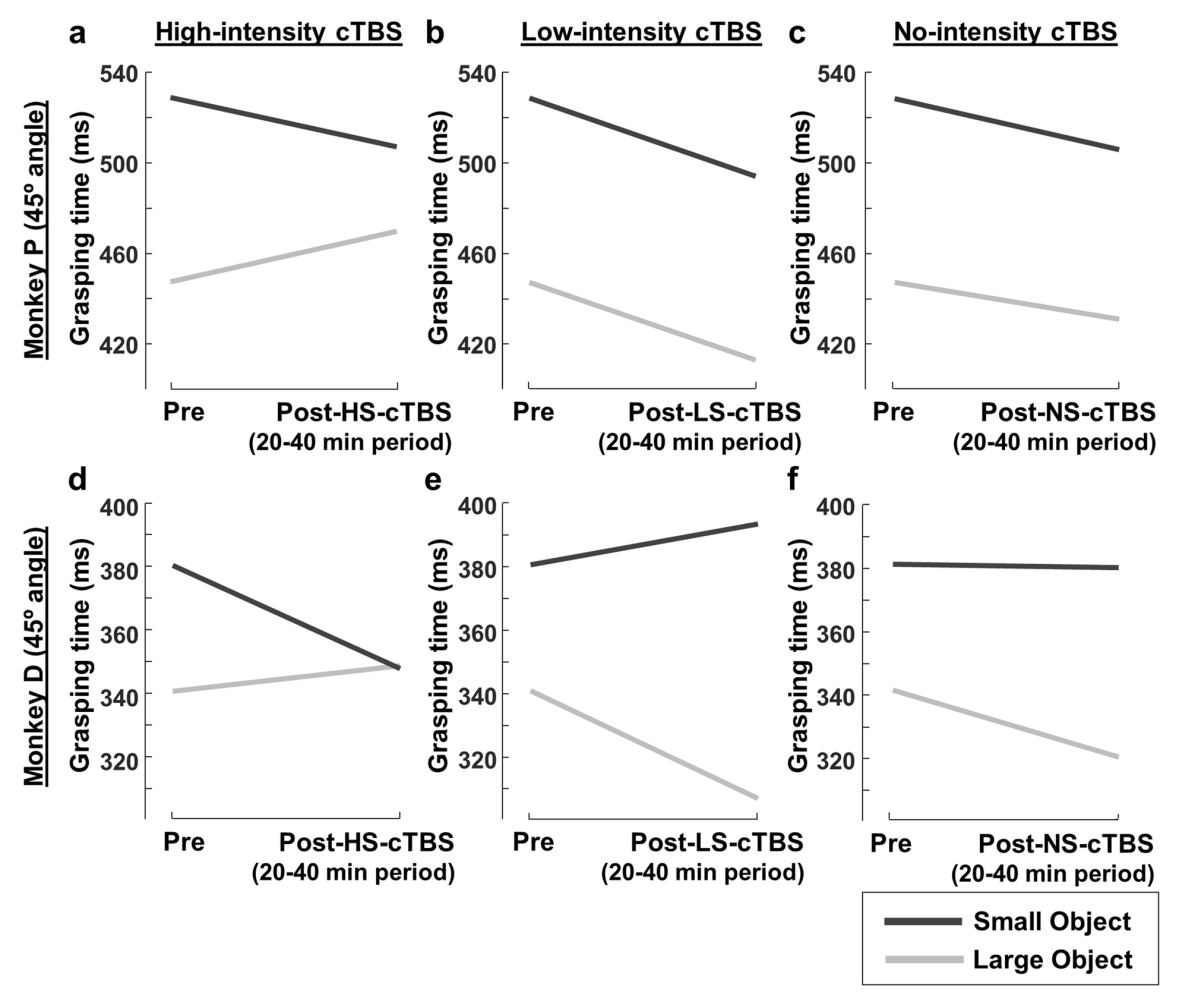
cTBS effect on Fitts’ law with a coil angle of 45 degrees. (**a**, **b**, **c**) Average pre-cTBS grasping time compared to the average GT at 20–40 min post-cTBS in monkey P, for the large (gray line) and small object (black line), with a coil angle of 45 degrees, for high-intensity (**a**), low-intensity (**b**) and no cTBS (**c**). (**d**, **e**, **f**) Pre/post-cTBS comparison for monkey D; same conventions as in (**a**, **b**, **c**).

To investigate to what extent cTBS disrupted Fitts’ law in our experiments, we compared the average pre-cTBS GT with the average GT at 20–40 min post-cTBS, an epoch in which the effects of cTBS in humans are maximal^8^ (Fig. 3). We chose this interval to relate to the human studies and to keep the same post-cTBS interval for the two animals, but comparing the pre-cTBS GTs with the GTs in the interval with the largest effect in each monkey yielded very similar results (two-way ANOVA with factors object and time epoch, *p* = 3.316e^−21^ for monkey D and *p* = 5.263e^−15^ for monkey P). In monkey P, the 82 ms GT difference between the large and the small object decreased to 37 ms after HS-cTBS (Fig. 3a; two-way ANOVA with factors *object* and *time-epoch (pre-post 20–40 min)*; F(1,4768) = 89.92, interaction effect, *p* = 3.795e^−21^, ${{\eta}}_{\mathrm{p}}^{2}$ = 0.019). This effect was even stronger in monkey D (Fig. 3d): the initial 40 ms GT difference between objects decreased after HS-cTBS to virtually identical average GT (-0.8 ms; two-way ANOVA, F(1,4834) = 91.49, interaction effect; *p* = 1.739e^−21^, ${{\eta}}_{\mathrm{p}}^{2}$ = 0.019). In contrast, LS-cTBS did not reduce the GT difference between the objects in monkey P (Fig. 3b; two-way ANOVA; F(1,4796) = 0, interaction effect, *p* = 1) and even increased in the case of monkey D (Fig. 3e; two-way ANOVA; F(1,5043) = 156.23, interaction effect, *p* = 2.505e^−35^, ${{\eta}}_{\mathrm{p}}^{2}$ = 0.030) the GT difference between the objects. NS-cTBS sessions showed similar effects as LS-cTBS sessions (interaction effect between object and time-epoch, F(1,5224) = 2.28, *p* = 0.131 for monkey P and F(1,4878) = 24.71, *p* = 6.89e-07 for monkey D).

**Figure 4:**
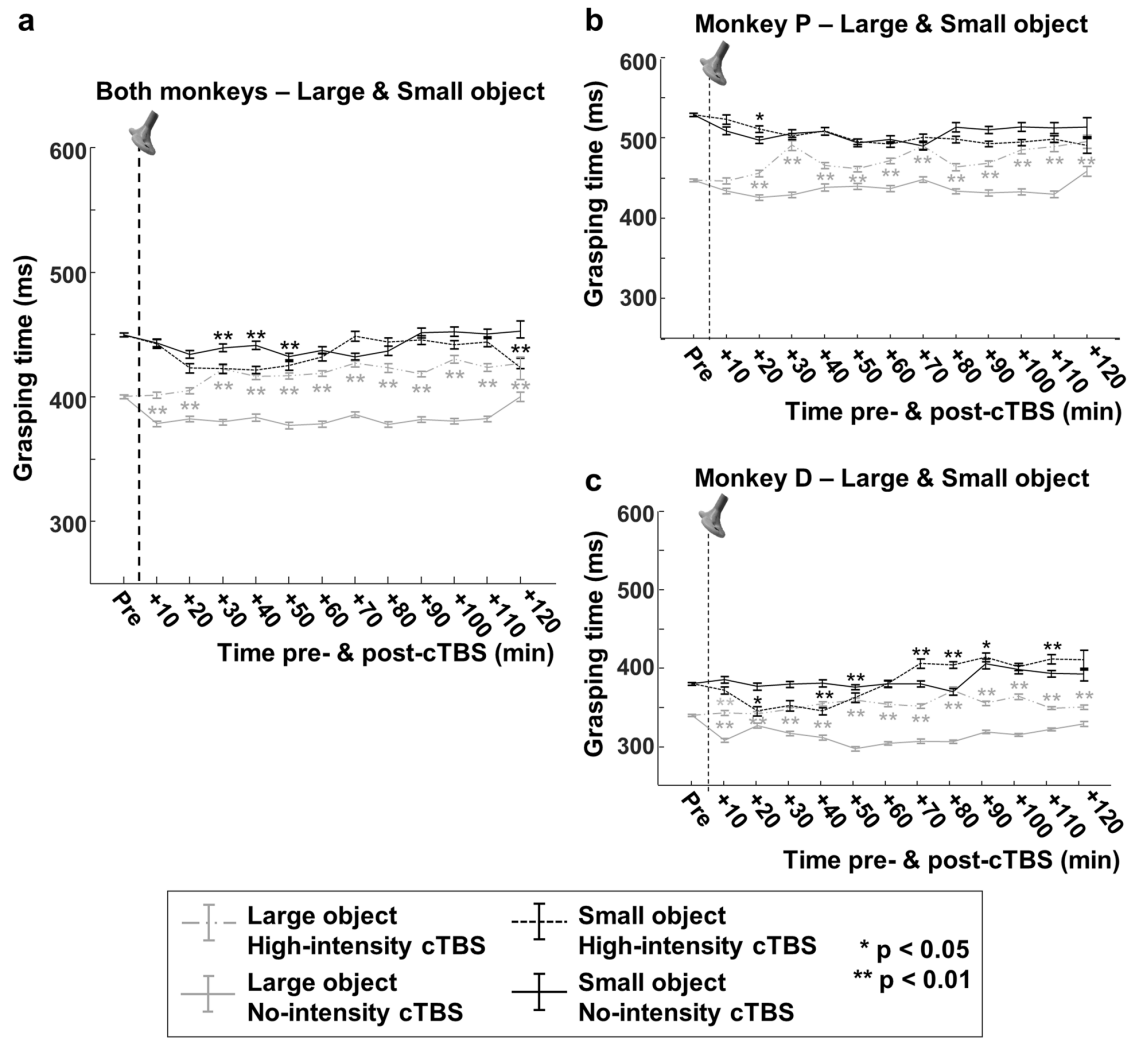
Overtime effects of cTBS on Fitts’ law. (**a**) Normalized grasping times of the two monkeys combined, when performing the visually-guided grasping task with the large versus the small object under different stimulation conditions: dashed grey line: large object/high-intensity cTBS; dashed black line: small object/high-intensity cTBS; solid grey line: large object/no cTBS; solid black line: small object/no cTBS. (**b**, **c**) Same data for the two monkeys separately ((**b**): monkey P, (**c**): monkey D). Asterisks indicate statistical strength (two-sided Wilcoxon Ranksum test, *: *p* < 0.05, **; *p* < 0.01; high-intensity cTBS time-epochs compared to no-intensity cTBS time-epochs). The vertical dashed line represents the stimulation time (cTBS).

To visualize the differential cTBS effect on the two objects over time, we combined the GT data of the two monkeys (Fig. 4a; see Fig. 4b,c for data of the individual animals). In the interval from 30 to 50 min post-cTBS, the GTs for the two objects were virtually identical (on average 4.4 ms difference), after which the difference partially reappeared, albeit to a much lesser degree (16 ms on average) compared to no-stimulation sessions (61 ms). Notice that the GT declined after 10 min in the no-stimulation condition for the large object (mainly caused by monkey D), after which the GT remained more constant over time. This drop in GT was most likely due to aspecific factors (motivation, becoming more engaged in the task) and was not observed after high-intensity cTBS. Overall, our results indicate that HS-cTBS applied over parietal cortex disrupts the neural control of visually guided object grasping: monkeys grasp large objects slower and small objects faster, so that the predictions derived from Fitts’ law become invalid or strongly reduced after cTBS. In addition, the effect on grasping behavior is clearly dose-dependent since low-intensity cTBS does not cause this effect, excluding non-specific TMS factors as potentially contributing to this phenomenon.

### Effect of coil-angle on grasping time

**Figure 5:**
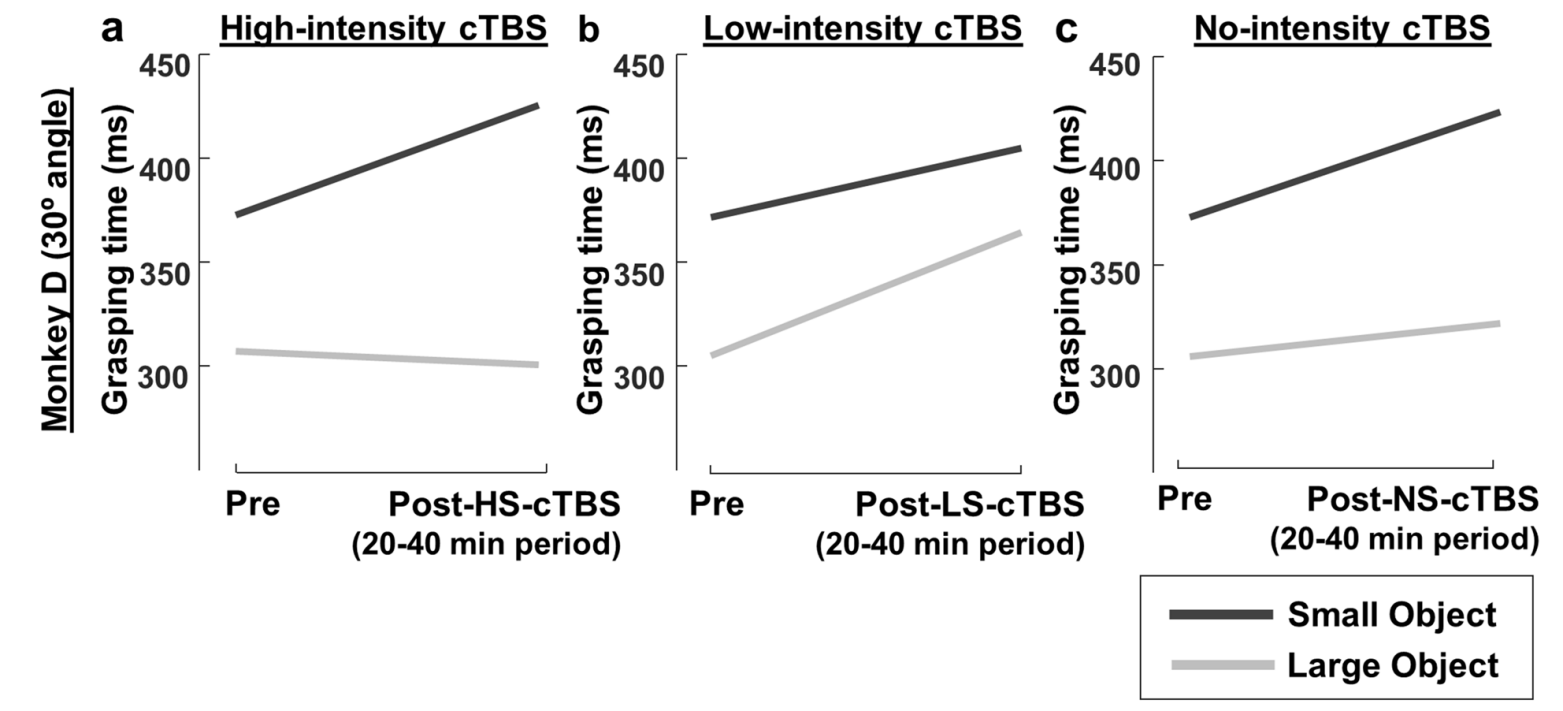
cTBS effect on Fitts’ law with a coil angle of 30 degrees. (**a**, **b**, **c**) Average pre-cTBS grasping time compared to the average GT at 20–40 min post-cTBS, for the large (gray line) and small object (black line), with a coil angle of 30 degrees, for high-intensity (**a**), low-intensity (**b**) and no cTBS (**c**), in monkey D.

**Figure 6:**
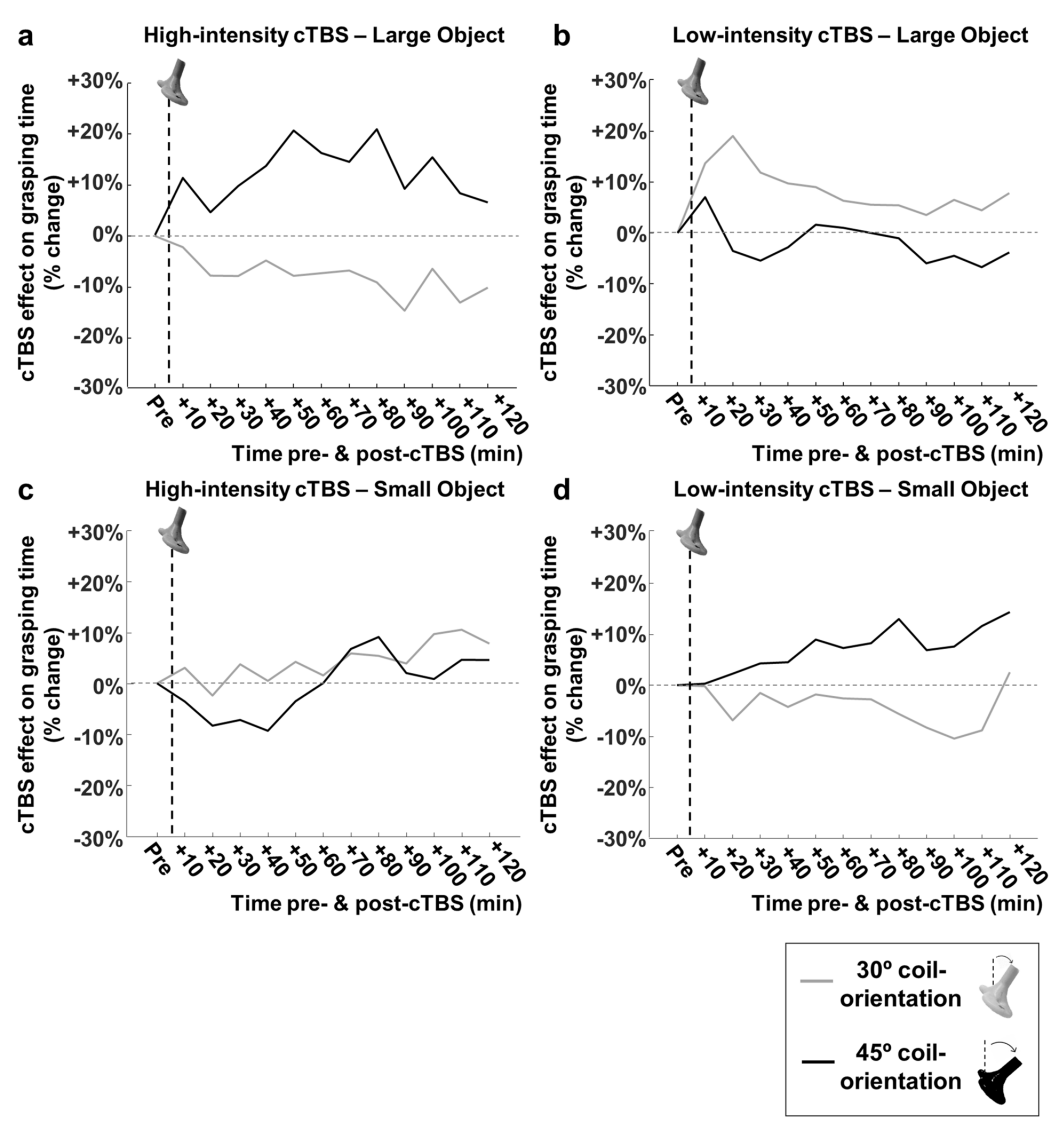
Coil angle and cTBS effect on grasping time. (**a**, **b**) Average percent change in grasping time (compared to no-stimulation) for high- (**a**) and low-intensity cTBS (**b**) when grasping the larger object, in monkey D. Black: 45-degree coil angle. Gray: 30-degree coil angle. The lighter dashed line (horizontal) represents the baseline grasping time. The thicker dashed line (vertical) indicates the stimulation time (cTBS). (**c**, **d**) Average percent change in grasping time (compared to no-stimulation) for high- (**c**) and low-intensity cTBS (**d**) when grasping the smaller object.

Our main experiments were targeting area PFG on the parietal convexity, an area that is part of the parieto-frontal network involved in object grasping. To test the spatial specificity of the cTBS effect on GT, we ran a control experiment in monkey D, in which we changed the angle of the guiding rods with respect to the vertical plane (30 degrees instead of 45 degrees) while preserving their anchoring positions on the skull. We then repeated the experiment with high-intensity, low-intensity and no-cTBS and the large and small objects. Figure 5 compares the average pre-cTBS GT with the average 20–40 min post-cTBS at the 30-degree angle. Surprisingly, cTBS applied with the coil at a 30-degree angle induced an effect opposite to cTBS applied with the coil oriented at 45 degrees. HS-cTBS caused an even larger difference in GT between the large and the small object (Fig. 5a; two-way ANOVA with factors *object* and *time-epoch*; interaction effect, F(1,4898) = 70.90, *p* = 4.878e^−17^, ${{\eta}}_{\mathrm{p}}^{2}$ = 0.014), similar to the effect of low-intensity cTBS at the standard 45-degree orientation in this monkey (Fig. 3e). Conversely, LS-cTBS tended to decrease the GT difference between the large and the small object (Fig. 5b; two-way ANOVA; F(1,4971) = 13.76, interaction effect, *p* = 0.0002,${{\eta}}_{\mathrm{p}}^{2}$ = 0.003). Thus, the cTBS effect on object grasping depended heavily on the orientation of the TMS coil, since a 15-degree difference in orientation was sufficient to induce opposite behavioral effects. Figure 6 summarizes the effect of coil angle on grasping performance. For both the large (Fig. 6a,b) and small objects (Fig. 6c,d), cTBS induced a significant difference (average percent change compared to no-stimulation) in GT, which sign depended on both the amount of stimulation induced (low- vs. high-intensity stimulation; Fig. 6b,d vs. Figure 6a,c) and the coil angle (45-degree vs. 30-degree coil position).

## Discussion

We applied a well-known repetitive TMS protocol (cTBS) over the parietal cortex of rhesus monkeys and observed robust behavioral effects on object grasping. The temporal dynamics of these cTBS behavioral effects resembled those described in human volunteers. Our cTBS protocol disrupted normal grasping in a dose-dependent manner, abolishing the difference in grasping time between large and small objects. Moreover, a 15-degree change in coil orientation was sufficient to induce opposite effects on grasping behavior.

The inferior parietal lobule consists of several cortical areas organized along a rostro-caudal axis. The most rostral (anterior) area, PF, contains many neurons responding to orofacial movements and stimulation, while neurons in the middle area PFG respond to hand movements and action observation (mirror neurons^20^), and the most caudal (posterior) area, PG (also known as area 7a), responds to arm movements^21^. Because visual fixation neurons are present in PG^22,23^ but not in PFG, and since previous extensive recordings in monkey P^15^ did not find visual fixation neurons, we conclude that our cTBS protocol effectively inactivated (part of) area PFG. Very few studies have investigated the effects of lesions or temporary interference of area PFG during grasping. Faugier-Grimaud et al.^24^ and Rushworth et al.^25^ observed deficits in reaching movements after lesions to the inferior parietal lobule, most likely including area PFG. Recently, we showed that single-pulse TMS applied over PFG at the lift of the hand caused longer grasping times^15^. Although in this previous study the TMS protocol investigated was different (single-pulse TMS) and only a single large object was tested, the magnitude of the observed effect was very comparable. The increase in GT for the large object with single-pulse TMS was + 66 ms compared to + 62 ms for the same monkey (monkey P) at the peak of the effect in the current study (cTBS). Thus, both types of TMS protocols indicate a causal role of PFG in object grasping during visual guidance.

Because we did not detect the time at which the hand touched the object, we could not dissociate the cTBS effect on the reaching phase from its effect on the grasping phase. Therefore, the longer GTs we measured after high-intensity cTBS may have originated from either a reaching deficit, a grasping deficit, or from both. The specificity of the cTBS effect for differently sized objects requiring the same reaching movement strongly suggests that cTBS over parietal area PFG disrupted the grasping phase of the action. However, since object size can also influence the reaching kinematics^26^, this problem can only be addressed with highly accurate tracking of the arm and hand, for example with markerless pose estimation^27^.

The absolute GTs in our monkeys varied strongly from session to session and even within sessions without stimulation (e.g. NS-cTBS data for the large object in monkey D.). This variability is not unexpected since the animals exhibit different levels of motivation between and within sessions. Therefore, we analyzed changes in GT after subtracting the average GT in the pre-cTBS interval, and compared these residual GT between the 3 levels of stimulation (high, low and no stimulation).

The temporal dynamics of the behavioral effects we observed overlapped very well with the known neurophysiological changes induced by cTBS. In human volunteers, the reduction in the amplitude of the MEP evoked over primary motor cortex is maximal after 20 min^8^ (for a cTBS application of 20 s). Moreover, in a parallel study^28^, we measured the reduction in neuronal excitability of area PFG during passive fixation using the same cTBS parameters as in the current study. The reduction in neuronal excitability was nearly maximal in the interval 20–30 min post-cTBS, and remained constant up to 60 min post-cTBS (a small number of neurons could also be recorded for 120 min post-cTBS without recovery to their baseline levels). Averaged over our two monkeys in the present study, the behavioral effect reached its maximum at 30 min post-cTBS, remained relatively constant after that up to 100 min post-cTBS, similar to the effect at the neuronal level.

It is noteworthy that we did not observe a general increase in the grasping time after cTBS, but rather a very specific increase in GT for the large object and a decrease in GT for the small object. An explanation of our results is offered by the original description of Fitts’ law. The reversible inactivation of PFG may have impaired visuomotor object processing (the *Width* term in the definition of the Index of Difficulty) while maintaining the estimation of distance (the *D* term, which was fixed in our experiments). Consequently, our large and small object were processed similarly, leading to similar grasping times. Arguing against this interpretation is our observation that we almost never encountered PFG neurons responding to the onset of light above the object^15,28^. Rather, almost all task-related neurons only started to modulate their activity after the hand had started to move towards the object. This suggests that our PFG reversible inactivation impaired at least part of the parietal inputs mediating visuomotor information to ventral premotor cortex (PMv), which consequently perturbed the normal interactions between PMv and primary motor cortex required for object grasping^29–31^.

The effects of LS-cTBS are more difficult to interpret. It is possible that LS-cTBS exerted a different effect on excitatory and inhibitory neurons in PFG compared to HS-cTBS. In line with this hypothesis, Romero, Davare et al.^15^ showed that single-pulse TMS affects both excitatory and inhibitory neurons, since the initial excitation of the large pyramidal neurons could be followed by temporary inhibition, which resulted most likely from the activation of nearby inhibitory interneurons. Therefore, it is conceivable that LS-cTBS shifted the balance of excitation and inhibition in PFG (e.g. more reduction in the excitability of inhibitory neurons and no effect on excitatory neurons), which may have caused opposite effects compared to HS-cTBS. In line with this idea, several studies have reported strong dose-dependent effects of cTBS on the MEP amplitude in humans^32,33^. Future studies should investigate in detail how LS-cTBS affects single-neuron activity.

Similar to LS-cTBS, changing the orientation of the TMS coil induced opposite effects compared to the standard experiment. It is difficult to identify which factors contributed to the observed phenomenon: a change in the induced electric field, an increase in the distance to the cortex or a shift in the anatomical area targeted (e.g. area 5 on the medial bank, or dorsal LIP on the lateral bank of the Intraparietal Sulcus). Since the behavioral effects were remarkably similar to the ones measured with LS-cTBS, the most parsimonious explanation is that the 30-degree coil orientation was less optimal for targeting PFG, thereby mimicking stimulation of the area at a low intensity. However, LS-cTBS with the 30-degree coil orientation tended to induce opposite effects on the GTs, which is difficult to reconcile with a pure effect of stimulation intensity and may suggest that we targeted different populations of neurons. Irrespective of the underlying cause, our control experiment illustrates clearly that relatively small changes in coil positioning may cause dramatic changes in behavioral effects, highlighting the focality of the technique, which undoubtedly contributes to the well-known interindividual variability of the cTBS effects in human volunteers^34–36^.

Finally, our data demonstrate the feasibility of implementing noninvasive neuromodulation techniques during behavioral testing in nonhuman primates. To bridge the gap between behavioral studies in human volunteers and invasive recordings in animals, it is essential to use identical tasks and techniques in the two species. Unlike human studies, the nonhuman primate model offers the possibility to detect novel behavioral effects and then test the underlying neural mechanisms using invasive recordings.

## Methods

### Animals and surgeries

We investigated cTBS effects on grasping performance using adult rhesus monkeys with prior, extensive experience in visually-guided grasping. Before the start of the experiments, two male rhesus monkeys (Macaca mulatta; monkey P, 10 kg; monkey D, 7 kg) were trained to sit in a primate chair. Each monkey then received a titanium head post, attached to the skull with ceramic screws and dental acrylic. All surgical procedures were performed under strict aseptic conditions and propofol anesthesia (10 mg/kg/h). All animal care and experimental procedures were conducted in accordance with the National Institutes of Health Guide for the Care and Use of Laboratory Animals and the EU Directive 2010/63/EU, and were approved by the Ethical Committee on animal experiments at KU Leuven.

### Estimation of the position of the TMS coil

To estimate the center of stimulation, we used anatomical Magnetic Resonance Imaging (MRI) and Computed Tomography (CT, only in monkey D). For each monkey, we first calculated the position of the coil over area PFG based on stereotactic coordinates, and then, implanted two guiding rods to the skull (over the right hemisphere in monkey P and over the left hemisphere in monkey D) using dental acrylic and under ketamine/medetomidine sedation. The use of these rods allowed a highly reproducible positioning of the TMS coil across sessions^15^.

**Figure 7:**
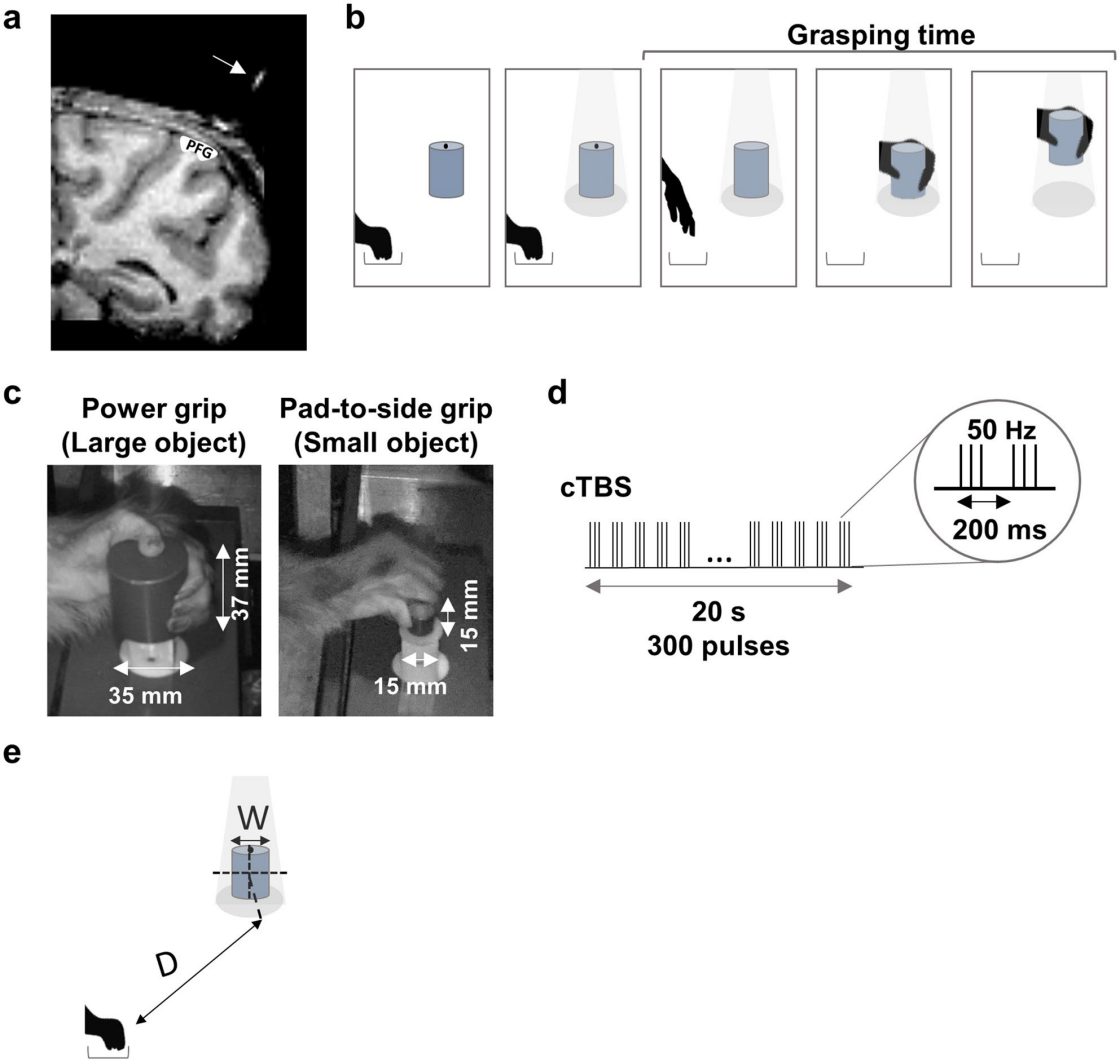
Experimental protocol. (**a**) Anatomical magnetic resonance image with the parietal area PFG indicated in white. The 25 mm figure-of-eight coil was positioned at a 45-degree angle with respect to the vertical. The white line (indicated by an arrow) represents the center of the TMS coil. (**b**) Visually-guided grasping task, which required the monkey to reach, grasp and lift an object after a go-signal (dimming of the red laser). (**c**) Grip types for the two cylindrical objects of different sizes used in the experiments. Grasping the large object (35 mm × 37 mm) elicited a power grip (whole-hand grasp), while the small object (15 mm × 15 mm) required a pad-to-side grip (involving exclusively the index finger and thumb). (**d**) Stimulation protocol. Our continuous Theta-Burst Stimulation (cTBS) protocol consisted of 50 Hz triplets of pulses applied every 200 ms. In total, 300 pulses were applied for 20 s. (**e**) Illustration of Fitts’ Law, stating that the time required to move to a target area is a function of the ratio between the distance to the target (D) and the width of the target (W).

In the main experiment, the rods were placed at a 45-degree angle with respect to the vertical plane. In one additional control experiment, the rods were angled at 30 degrees with respect to the vertical plane while preserving the antero-posterior and medio-lateral position, which allowed direct comparisons of the two coil orientations. This 30-degree angle was chosen because we wanted to preserve the overall location of the coil above parietal cortex and for practical reasons, since a larger coil angle was impossible to achieve in our setup. After the implantation of the rods, we obtained an anatomical MRI with a custom-built MRI-compatible dummy coil positioned over the rods (Fig. 7a). This dummy coil had identical dimensions to the actual TMS coil, but contained a central adapter holding a glass capillary, which was filled with a 2% copper sulphate solution to obtain a clear image of the center of the coil and its projection into the brain. With this technique, we were able to determine accurately the position, angle and distance between the coil and the brain following each rod implantation (Fig. 7a). For both monkeys in this study, we used these MR images to verify that the TMS coil was placed over parietal area PFG at a distance of approximately 15 mm from the parietal convexity. In this position, the coil was oriented to induce a postero-anterior (PA) current over PFG. During the experiments, a metal arm provided additional support to the coil and cable.

### Visually-guided grasping task

During the experiments, the monkey sat in a primate chair with its head fixed. The ipsilateral hand was lightly restrained to enforce grasping with the contralateral hand. We monitored the position of the right eye with an infrared eye-tracking camera (EyeLink 500). Both monkeys were trained to hold their gaze in an electronically defined fixation window (+ /− 2.5-degree window), centered around the object. To start the trial (Fig. 7b), the monkey had to hold his hand in a resting position. After a variable time (inter-trial interval: 2000–3000 ms), a red laser was projected on top of the object. If the animal maintained its gaze within the electronically defined fixation window for 500 ms, an external light source illuminated the object. Following a variable delay (900–1100 ms), the red laser light switched off, which served as a go-signal to lift the hand from the resting position, and reach, grasp, lift and hold the object for 500 ms. When the monkey performed the sequence correctly, it received a drop of apple juice as reward. Fiber optic cables located at the resting position and under the to-be-grasped object detected both the lift of the hand and the pull of the object. For all stimulation sessions (low- and high-), we also obtained video recordings of the task performance which allowed the offline evaluation of a potential grasping deficit (such as a deficit in hand preshaping).

To compare the effect of cTBS on different grip types, we used two cylindrical objects: a large object (diameter 35 mm, height 37 mm; Fig. 7c, left image) and a small object (diameter 15 mm, height 15 mm; Fig. 7c, right image). The two objects required two types of handgrip: a power grip for the large object (whole-hand grasp) and a pad-to-side grip (a variant of the precision grip) for the small object (involving exclusively the index finger and the thumb). The objects were positioned at the same distance from the monkey approximately 20 cm away from the hand at the resting position. In each experimental session, only one of the objects was presented, and the sessions were randomly interleaved. Prior to the experiments, we conducted a baseline study (4 sessions per object; see Table 1 for summary) to quantify the normal grasping performance, i.e. without TMS, of each monkey with the two objects (power vs. pad-to-side grip).

### cTBS paradigm

Following the baseline measurements, we applied cTBS using a Magstim Rapid Stimulator (Magstim, UK) and a custom-built figure-of-eight coil for animal use (D25 mm; 55 mm external diameter, similar to Romero et al.^15^). The stimulation paradigm consisted of 50 Hz triplets repeated at 200 ms intervals (in total 300 pulses) for a duration of 20 s (as in Huang et al.^8^, Fig. 7d). We collected behavioral data for the two objects in three stimulation conditions: no-stimulation (NS) with the TMS coil positioned on the skull, low-intensity stimulation (LS-cTBS; at 40% of the resting Motor Threshold, rMT) and high-intensity stimulation (HS-cTBS; at 80% of the rMT). The rMT was the minimal intensity that evoked a twitch in the hand contralateral to the stimulated primary motor cortex. All six conditions (2 × 3 factorial design; large and small objects, and no-, low- and high-intensity stimulation) were pseudo randomly interleaved across sessions. For each condition, we collected between 1705 and 3314 correct trials (Table 2).

**Table 2:** Total number of sessions and correct trials collected for both the cTBS (monkey P & D, coil-angle at 45 degrees) and the control experiment (monkey D, coil-angle at 30 degrees).

Object type	High-intensity cTBS			Low-intensity cTBS			No-intensity cTBS		
	Nr of sessions	Nr of correct trials	Total number of trials	Nr of sessions	Nr of correct trials	Total number of trials	Nr of sessions	Nr of correct trials	Total number of trials
Monkey P (45°)									
Large object	3	2147	2398	3	2212	2547	4	3314	3604
Small object	3	2380	2778	3	2175	2476	3	2219	2566
Monkey D (45°)									
Large object	3	2405	2761	3	2606	2891	3	2759	3149
Small object	3	2391	2702	3	3145	3469	3	2744	3102
Monkey D (30°)									
Large object	3	2425	2766	3	2771	3044	2	1705	1959
Small object	3	2481	2715	3	2834	3011	3	2213	2643

In these experiments, the monkeys were exposed to 6 different conditions: 2 grasping objects (large, small) × 3 stimulation conditions (high-, low- or no-intensity cTBS).

For every session, the monkey first performed the grasping task during a baseline block of 20 min. Next, we applied low- (40% of the rMT, LS) or high-intensity (80% of the rMT, HS) cTBS offline for 20 s. After stimulation, the monkey continued grasping for up to 120 min. LS- and HS-cTBS sessions were interleaved with NS sessions, where cTBS was not applied. Both monkeys showed no side effects during or after cTBS stimulation and performed the grasping task without signs of distress.

At the end of these sessions, in monkey D, we repeated the experiment using a different coil orientation. To do this, we adjusted the rods orientation with respect to the vertical plane (30 degrees instead of 45 degrees) while preserving their center position on the skull (control experiment, Table 2).

### Statistical analysis

All data were analyzed in MATLAB (R2017A, MathWorks, Massachusetts, USA). To quantify the difference between grasping performance with the large and the small object, we calculated an index of difficulty (ID) for the two objects^37^. Inspired by Shannon’s Theorem 17^38^, Fitts’ law states that the time required to move to a target area is a function of the ratio between the distance to the target (D) and the width of the target (W) (Fig. 7e). Fitts’ index of difficulty (ID, in bits, Eq. (1)) is defined as:

1 $$ {\text{ID}} = \log_{2} \left( {{\text{D/W}} + 1} \right) $$

In this formulation, the distance to the center of the target is considered as the signal and the object width is considered as noise (uncertainty). In our experiments, the ID was equal to 2.75 for the large object and 3.84 for the small object, which yielded a difficulty ratio of 1.40.

For each trial, we calculated the time elapsed between the start of the hand movement (i.e. the moment that the hand did not interrupt the light produced by the fiber-optic cables at the resting position) and the lift of the object (grasping time, GT). In the baseline experiment, we compared the average GT required to grasp the large and small object with a two-sided Wilcoxon signed-rank test. Because of the inherent intersession variability in grasping behavior in both monkeys, GTs were normalized by subtracting the average GT of all pre-cTBS trials in that session from each post-cTBS trial. This subtraction implies that we analyzed changes in GT (pre- vs. post-cTBS GT) for every NS, LS-cTBS and HS-cTBS sessions. When plotting the data, we added the average pre-cTBS GT collected across trials and sessions to each data point, so that the differences in GT between the large and the small object were visible.

To quantify the effect of cTBS across stimulation intensities and over time, all sessions of the same stimulation condition were combined and divided in 10 min epochs. We calculated two-sided Wilcoxon signed-rank tests (corrected for multiple comparisons, Bonferroni corrected) to compare each NS time interval with the corresponding time interval after LS- or HS-cTBS. Additionally, we computed the number of motor errors (i.e. errors between the go-cue and the pull of the object) committed in these 10 min epochs (Table 3). The numbers of trials used in these tests are provided in Tables 1 and 2.

**Table 3:** Percentage of errors made with the large and small object for the high-, low- or no-intensity cTBS conditions (for each monkey, 45-degrees vs 30-degrees).

Object type	Stimulation condition	− 20 min	− 10 min	10 min	20 min	30 min	40 min	50 min	60 min	70 min	80 min	90 min	100 min	110 min	120 min
Monkey P (45°)															
Large object	HS-cTBS	11.7	13.9	13.6	7.8	6.0	9.0	12.1	11.0	8.7	10.0	10.3	14.3	7.9	11.7
	LS-cTBS	20.1	16.5	16.8	9.5	12.2	8.4	12.9	13.9	9.6	16.7	15.6	11.4	18.2	20.1
	NS-cTBS	10.0	8.9	7.8	7.6	5.5	8.0	4.7	7.4	6.2	8.8	10.3	11.3	2.3	10.0
Small object	HS-cTBS	19.6	15.4	13.9	13.8	14.7	13.8	11.7	12.1	16.8	12.0	13.7	18.5	17.8	19.6
	LS-cTBS	20.9	16.6	11.4	15.7	9.8	15.0	11.7	9.6	10.0	6.9	10.6	13.0	11.8	20.9
	NS-cTBS	23.7	13.6	14.6	10.7	13.5	14.0	12.6	12.4	15.2	9.4	19.5	13.5	12.5	23.7
Monkey D (45°)															
Large object	HS-cTBS	15.4	10.8	8.5	8.0	5.6	7.6	16.0	18.5	18.1	15.5	16.6	16.9	15.7	15.4
	LS-cTBS	7.2	4.0	4.4	8.5	8.0	8.5	13.0	12.0	10.4	12.6	10.6	13.6	20.8	7.2
	NS-cTBS	6.3	12.1	14.0	11.8	11.3	11.7	9.8	10.3	12.3	11.3	13.4	15.3	19.3	6.3
Small object	HS-cTBS	11.7	9.8	12.9	11.2	10.1	12.0	7.6	6.3	9.0	15.7	15.4	17.3	26.3	11.7
	LS-cTBS	10.4	12.4	7.6	6.9	9.9	8.8	10.2	10.7	6.9	7.5	7.5	10.4	17.6	10.4
	NS-cTBS	11.3	8.1	11.8	10.8	12.4	12.7	9.4	7.0	15.1	15.7	9.3	16.5	10.4	11.3
Monkey D (30°)															
Large object	HS-cTBS	17.3	11.2	11.9	14.5	14.2	8.3	13.1	10.1	11.7	17.6	11.9	11.0	14.0	17.3
	LS-cTBS	14.6	5.5	12.5	8.8	7.9	7.8	9.0	8.1	9.2	8.5	9.4	12.2	10.2	14.6
	NS-cTBS	17.8	12.9	13.5	13.2	10.6	15.5	9.8	15.4	9.1	15.8	14.8	11.1	0.0	17.8
Small object	HS-cTBS	10.4	10.6	7.7	9.0	6.3	9.1	7.9	8.3	7.3	9.3	11.2	9.0	11.5	10.4
	LS-cTBS	16.8	14.0	13.4	8.8	10.6	9.1	9.5	8.8	9.5	11.6	11.6	13.5	24.4	16.8
	NS-cTBS	18.7	17.0	15.3	13.6	16.0	15.1	15.5	16.0	11.5	14.9	7.9	6.3	16.7	18.7

To evaluate the overall effect of stimulation intensity on GT for the entire 120 min post-cTBS interval, we performed a Kruskal–Wallis one-way ANOVA across stimulation intensities and separately for each animal. To quantify the effect of cTBS over time, we calculated repeated measures ANOVA on the GTs with *time* (pre to post 120) and *stimulation intensity* (NS, LS-cTBS, HS-cTBS) as factors. Finally, to test the difference between the cTBS effect observed across objects in the interval 20–40 min post cTBS, we calculated a two-way ANOVA for each stimulation condition, with the factors *object* (small vs. large) and *time-epoch* (pre-cTBS vs the 20–40 min post-cTBS interval).

## Data Availability

The datasets generated during and/or analyzed during the current study are available from the corresponding author on reasonable request.
